# The relationship between trajectories of renal oxygen saturation and acute kidney injury: a prospective cohort study with a secondary analysis

**DOI:** 10.1007/s40520-024-02701-1

**Published:** 2024-02-21

**Authors:** Chang Liu, Xiaoxiao Wang, Wenzhu Shi, Yao Yu, Xiaoling Sha, Peipei Wang, Siyi Yao, Zhao Li, Yanhong Liu, Jiangbei Cao, Hao Li, Weidong Mi

**Affiliations:** 1 Department of Anesthesiology, The First Medical Center, Chinese PLA General Hospital, Beijing, 100853 China; 2 Medical School of Chinese PLA General Hospital, Beijing, China; 3 National Clinical Research Center for Geriatric Diseases, Chinese PLA General Hospital, Beijing, China; 4 Research Center of Clinical Epidemiology, Peking University Third Hospital, Beijing, 100191 China

**Keywords:** Older patients, Renal tissue oxygen saturation, Trajectory, Near-infrared spectroscopy

## Abstract

**Background:**

Acute kidney injury (AKI) is a major postoperative consequence, affecting prognosis of older patients. Effective prediction or intervention to predict or prevent the incidence of AKI is currently unavailable.

**Aims:**

Dynamic changes of renal tissue oxygen saturation (RSO_2_) during surgery process are understudied and we intended to explore the distinct trajectories and associations with postoperative AKI.

**Methods:**

This was a secondary analysis including data for older patients who underwent open hepatectomy surgery with informed consent. Latent class mixed models (LCMM) method was conducted to generate trajectories of intraoperative renal tissue RSO_2_ through different time points. The primary outcome was postoperative 7-day AKI. The univariate and multivariate regression analysis were performed to identify the relationship between distinct trajectories of renal tissue RSO_2_ and the risk of AKI. Meanwhile, the prediction efficacy of renal tissue RSO_2_ at different time points was compared to find potential intervention timing.

**Results:**

Postoperative AKI occurred in 14 (15.2%) of 92 patients. There are two distinct renal tissue RSO_2_ trajectories, with 44.6% generating "high-downwards" trajectory and 55.4% generating "consistently-high" trajectory. Patients with "high-downwards" trajectory had significantly higher risk of postoperative AKI than another group (Unadjusted OR [Odds Ratio] = 3.790, 95% CI [Confidence Interval]: 1.091–13.164, *p* = 0.036; Adjusted OR = 3.973, 95% CI 1.020–15.478, *p* = 0.047, respectively). Predictive performance was 71.4% sensitivity and 60.3% specificity for "high-downwards" trajectory of renal tissue RSO_2_ to identify AKI. Furthermore, the renal tissue RSO_2_ exhibited the lowest level and the best results in terms of the sensitivity during the hepatic occlusion period, may be considered as a "time of concern".

**Conclusions:**

Older patients undergoing hepatectomy may show high-downwards trajectory of renal tissue RSO_2_, indicating a higher risk of AKI, and the lowest level was identified during the hepatic occlusion period. These findings may help to provide potential candidates for future early recognition of deterioration of kidney function and guide interventions.

**Supplementary Information:**

The online version contains supplementary material available at 10.1007/s40520-024-02701-1.

## Introduction

Acute kidney injury (AKI) is a major postoperative consequence, characterized by a rapid decline of renal function within a short period [1], with more than 5000 cases per million people, resulting in more than 1.7 million deaths per year [2]. The incidence of AKI after liver resection ranged from approximately 10% to 16% and even up to 21.6% after major hepatectomy [3, 4]. AKI may lead to chronic kidney disease (CKD) and end-stage renal disease (ESRD) even after renal function becomes its normal baseline level [5, 6]. Older patients with aging kidneys are at greater risk for severe consequences postoperatively, and AKI episodes may accelerate renal disease and even progression to mortality [7, 8]. Thus, it is imperative to identify the potential methods for early diagnosis and prognostic predictors in advanced-age patients for targeted implementation of renal protectants.

Near-infrared spectroscopy (NIRS) is a noninvasive technique that determines renal tissue regional oxygen saturation (RSO_2_) by measuring the relative concentrations of oxygenated and deoxygenated hemoglobin within a local tissue area and identifies renal hypoxia and hypoperfusion in a real-time manner [9]. It was first applied in pediatric surgery, indicating low renal tissue RSO_2_ values were correlated positively to peak serum creatinine levels [10], negatively to estimated glomerular filtration rate (eGFR) [11], and increased risk of developing AKI [10]. Recent studies with the application of NIRS found that persistent low renal tissue RSO_2_ can predict postoperative renal dysfunction among adult patients who underwent cardiac surgery [12, 13]. Our latest study demonstrated significant correlations between intraoperative renal oxygen desaturation and postoperative AKI [14], suggesting that monitoring renal oxygenation and perfusion using NIRS was a potential predictor of AKI. However, as a kind of continuously measured data, current studies did not profile the dynamic changes in the intraoperative renal tissue RSO_2_ during the entire course of surgery. Also, which time point was not determined as the optimal timing to guide intervention in the further experiment.

Therefore, the present study aimed to identify the typical renal tissue RSO_2_ trajectories, the key time points, and its association between trajectories and adverse renal outcomes in older patients after liver resection to assist early identification of AKI and improve the outcomes among surgical patients.

## Materials and methods

### Study design and population

Our study was a prospective, observational cohort study with secondary analysis. In the parent study, patients older than 60 years who underwent elective open hepatectomy between September 2020 and October 2021 in our hospital were included. Patients were excluded if they had one of the following criteria, patients who underwent liver transplantation, body mass index (BMI) exceeding 30 kg/m^2^, renal depth exceeding 4 cm measured from the capsule of the kidney to the skin surface by ultrasonographic examination, renal insufficiency (defined as an eGFR of < 60 mL/min/1.73 m^2^), surgery cancelled or changed and no informed consent. To further explore the dynamic changes of renal tissue RSO_2_, the secondary analysis established other inclusion criteria of with the process of hepatic inflow occlusion and with at least 300 measurements of renal tissue RSO_2_ to obtain sufficient statistics. Written informed consent was obtained from all eligible subjects or their family members in the parent study. Our primary study was also in line with the CONSORT statement and was approved by the Ethics Committee Board of the First Medical Center of Chinese PLA General Hospital (No. S2020–406–01). The trial was registered at clinicaltrials.gov (NCT04967105).

### Anesthetic and surgical procedures

Preoperative abstinence from drinking for 4 h and eating for 8 h. Standard monitoring was performed upon admission, including carrying out of electrocardiogram (ECG), noninvasive blood pressure (NIBP) measurement, pulse oximetry (SpO_2_), and bispectral index (BIS). Peripheral venous access and peripheral arterial cannulation were required before the induction of anesthesia. Drugs for inducing anesthesia were propofol (2 mg/kg), sufentanil (0.3 μg/kg), and rocuronium (0.6 mg/kg). Anesthesia was maintained with intravenous administration of propofol, and remifentanil supplemented with sevoflurane inhalation. The minimum alveolar concentration (MAC) value of sevoflurane was maintained < 1 and propofol (1.5–2.5 mg/kg/h) and remifentanil (8–10 μg/kg/h) were continuously administered to maintain a BIS of 40–60. Lung-protective ventilation strategy was maintained after tracheal intubation, with a tidal volume of 6–8 mL/kg, a respiratory rate of 10–14/min, an inspiratory to the expiratory ratio of 1:2, and an oxygen content of 60% of the inhaled gas. The fresh gas flow rate was 2 L/min to maintain PaCO_2_ between 35 and 45 mmHg. Following induction of anesthesia, an L-type incision on the right upper abdomen was made. To reduce intraoperative bleeding, the intermittent Pringle maneuver was used systematically with clamping for 15 min followed by a 5 min open period. After the removal of the tumor and hemostasis of the surgical field, a drainage tube was placed, and the incision was sutured layer by layer. All operations were performed by a team of surgeons specializing in hepatobiliary surgery.

### NIRS measurements

Before anesthesia induction, the bilateral sensors were placed on each side of the kidney to record renal tissue RSO_2_ using a NIRS monitor (INVOS 5100C Regional Oximeter; Medtronic, Minneapolis, MN, USA). Guiding with an ultrasound, the sensors were placed at the intercostal space, below either the 10th or 11th rib, on the posterior axillary line. Renal depth was measured manually as the distance from the skin surface to the renal capsule along the long axis and recorded while the NIRS was not used in patients whose renal distance reached 4 cm. Baseline renal tissue RSO_2_ was determined as the mean value during the first 3 min period after entering the operation room while the patients were breathing room air. Renal tissue RSO_2_ was continuously monitored every 30 s on both sides throughout the surgery. Renal tissue RSO_2_ during the critical moments of surgery (induction of anesthesia, initiation of surgery, initiation of hepatic vascular clamping, 5 min after clamping, 10 min after clamping, hepatic vascular release, 10 min after release, 20 min after release, 30 min after release, end of the surgery, and end of anesthesia) were determined as the mean value in both sides when specific surgical procedures were performed.

### Surgery outcomes

The primary outcome was the incidence of postoperative 7-day AKI. The kidney disease: improving the global outcome (KDIGO) criteria was used to assess the development of AKI. AKI was diagnosed by a 50% increase of serum creatinine from preoperative baseline level within 7 days after surgery or an increase of over 26.5 μmol/L (0.3 mg/dl) within 48 h. The criterion for urine output for ascertaining AKI was not elected as it was disturbed by the diuretic use and inaccurate recording in the ward. The secondary outcomes were the occurrence of other complications including infection, prolonged mechanical ventilation, surgery-related complications (bleeding, reoperation, and pancreaticoduodenectomy), hydrothorax and ascites, and admission to an intensive care unit (ICU), hospital length and in-hospital death.

### Data collection

Baseline characteristics, including age, sex, BMI, age-adjusted Charlson comorbidity index (ACCI), history of hypertension, diabetes mellitus, American Society of Anesthesiologists (ASA) classification, preoperative serum creatinine level, and red blood cell (RBC) count from the last examination before surgery, were collected. Intraoperative data, including duration of operation, estimated blood loss, urine output, and fluid transfusion were retrieved. Early postoperative complications were also recorded.

### Statistical analysis

The latent class mixed model (LCMM) was conducted using the R package lcmm, a method that classified individuals into groups or latent classes with similar trajectory patterns [15]. The latent classes were determined based on intraoperative renal tissue RSO_2_ at the twelve critical time points of surgery (baseline, induction of anesthesia, initiation of surgery, initiation of hepatic vascular clamping, 5 min after clamping, 10 min after clamping, hepatic vascular release, 10 min after release, 20 min after release, 30 min after release, end of surgery, and end of anesthesia). The optimal number of latent classes was determined according to the Akaike information criterion (AIC), Bayesian information criterion (BIC), sample size adjusted BIC (SABIC), and clinical judgment. Each participant was assigned to the latent class with the highest membership probability.

Continuous variables were reported as mean ± standard deviation (SD) or median (25% and 75% quartiles), and compared between groups (e.g., different renal tissue RSO_2_ trajectories) using an unpaired Student’s *t*-test or Mann–Whitney *U*-test. Categorical data were presented as proportion and compared between groups using a *χ*^2^ test or Fisher’s exact test. Multiple logistic regression was performed to find the association between renal tissue RSO_2_ trajectories and AKI while adjusting for potentially confounding variables. The sensitivity and specificity were calculated to evaluate the ability of renal tissue RSO_2_ trajectories and renal tissue RSO_2_ at each time point in predicting AKI.

Statistical analyses were performed using R statistical software (v.4.2.0; R Core Team 2022). A *p*-value of < 0.05 was considered statistically significant.

## Results

**Fig. 1:**
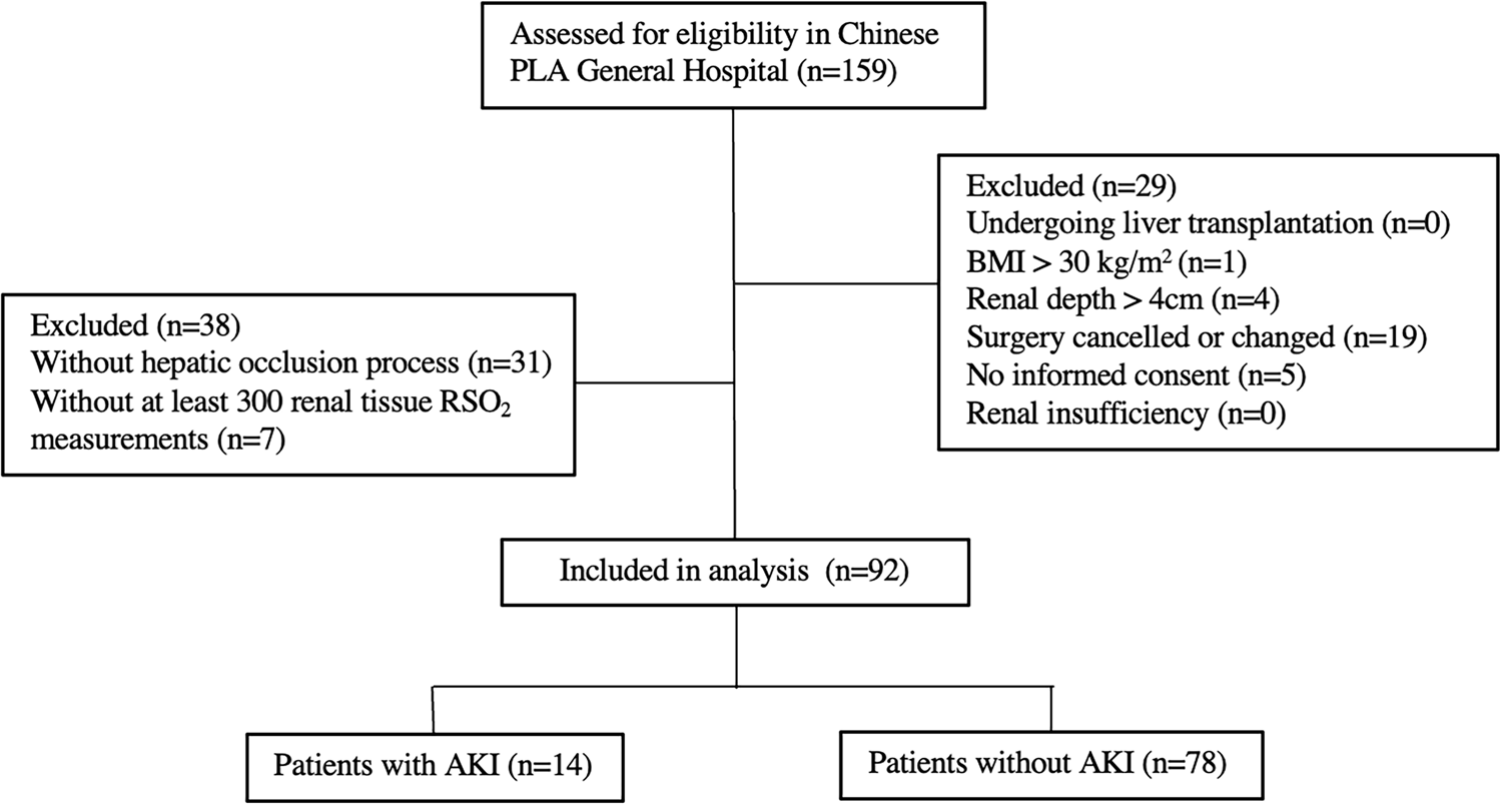
Patient selection flowchart

Among patients who underwent liver resection using hepatic occlusion technique at our hospital between September 2020 and October 2021, 92 patients met our inclusion criteria and included in the analysis. The flowchart of the patient selection was depicted (Fig. 1). Among the selected 92 patients, approximately 77.2% (71 of 92) patients were males, 23.9% (22 of 92) patients were ≥ 70 years old, and 56.5% (52 of 92) patients had overweight (BMI of ≥ 24 kg/m^2^) but with a renal depth of less than 4 cm.

**Fig. 2:**
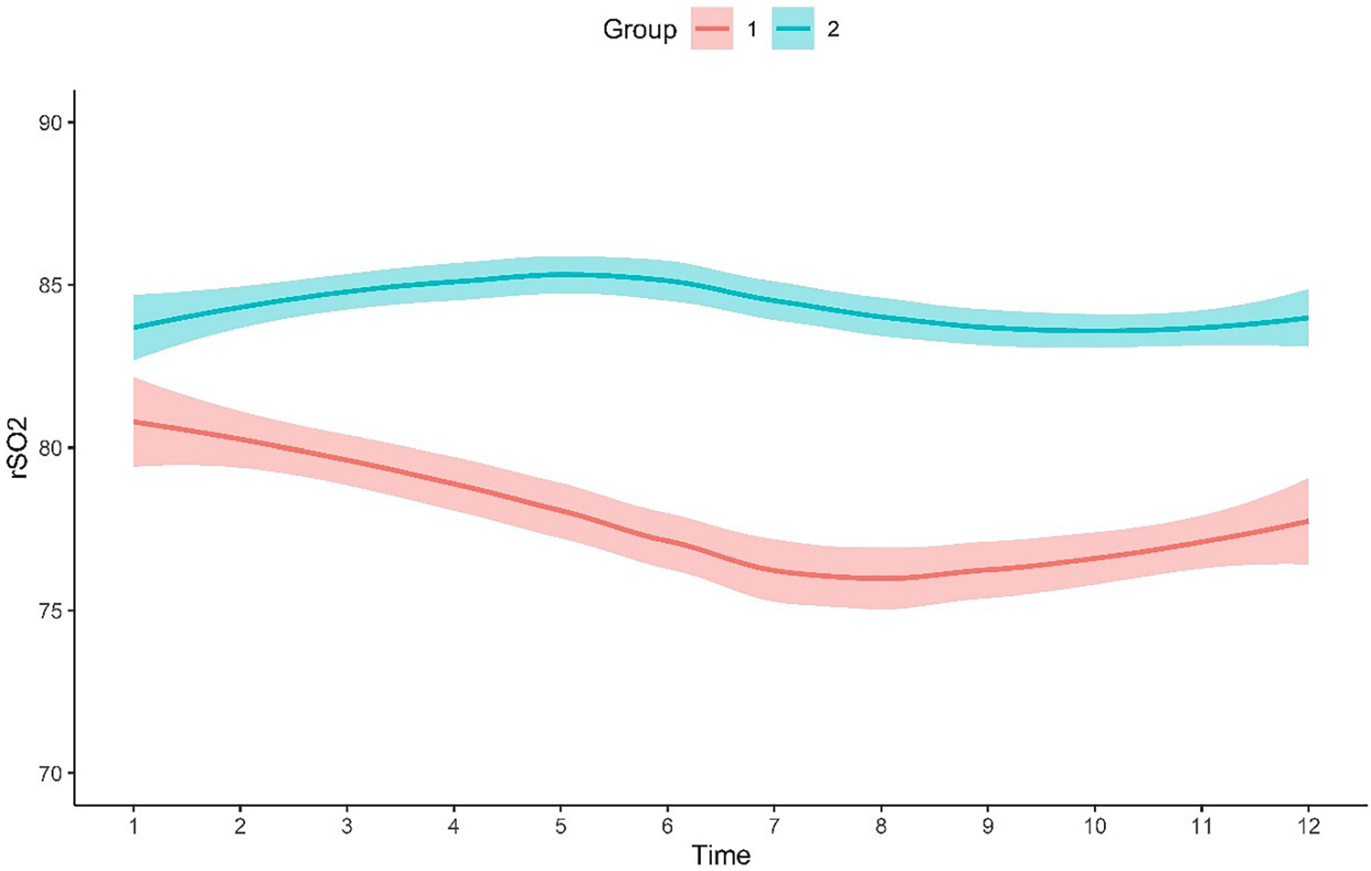
The different trajectories of renal tissue RSO_2_. Group 1 represents a "high-downward" trajectory, and Group 2 represents a "consistently-high" trajectory

LCMM were fitted with up to four latent classes. The two-class model was retained for the final output, depending on the statistical and clinical criteria. Figure 2 shows the trajectories of intraoperative renal tissue RSO_2_ change. Of all 92 patients, 41 (44.6%) patients showed a "high-downward" trajectory (group 1), and 51 (55.4%) fell into the "consistently-high" category (group 2). And the lowest renal tissue RSO2 was at the period of hepatic occlusion. Table 1 shows the baseline characteristics and intraoperative data of patients with distinct renal tissue RSO_2_ trajectories. Patients’ demographic characteristics were similar between the groups. Clinical data were also similar between the groups, including ACCI, history of hypertension, diabetes mellitus, ASA grade, preoperative serum creatinine level, and RBC count. Both groups did not differ in the duration of operation, colloid volume, crystal volume, and estimated blood loss. Patients in the "high-downward" trajectory (group 1) had significantly reduced urine output than those in the "consistently-high" trajectory (group 2).

**Table 1:** Baseline characteristics and intraoperative data in patients with distinct renal tissue RSO_2_ trajectories

Characteristics	"High-downward" group (*n* = 41)	"Consistently-high" group (*n* = 51)	*t/z/χ*^2^	*p*-value
Preoperative				
Age ≥ 70 y, *n* (%)	11 (26.8)	11 (21.6)	0.346	0.557
Gender, n(%)			0.103	0.749
Male	31 (75.6)	40 (78.4)		
Female	10 (24.4)	11 (21.6)		
BMI ≥ 24 kg/m^2^, *n* (%)	20 (48.8)	32 (62.7)	1.804	0.179
ACCI, median (IQR)	8 (7, 9)	8 (7, 9)	1.099	0.272
Hypertension, *n* (%)	16 (39.0)	21 (41.2)	0.044	0.834
Diabetes, *n* (%)	9 (22.0)	14 (27.5)	0.367	0.545
ASA, *n* (%)			–	0.571
I	1 (2.4)	0 (0)		
II	34 (82.9)	42 (82.4)		
III	6 (14.6)	9 (17.6)		
Serum creatinine (μmol/L), mean ± SD	69.8 ± 12.5	73.9 ± 14.5	–1.430	0.156
RBC count (10^12^/L), mean ± SD	4.2 ± 0.6	4.4 ± 0.5	–1.813	0.073
Intraoperative				
Surgery duration (min), median (IQR)	275 (220, 370)	260 (234, 345)	0.020	0.984
Colloid volume (mL), median (IQR)	500 (500, 500)	500 (500, 1000)	–1.448	0.148
Crystal volume (mL), median (IQR)	2200 (2100, 2600)	2100 (1600, 2600)	1.576	0.115
Urine volume (mL), median (IQR)	200 (100, 500)	350 (200, 700)	–2.257	0.024
EBL (mL), median (IQR)	200 (100, 400)	200 (100, 325)	0.562	0.574

RSO_2_: regional oxygen saturation; BMI: body mass index; ACCI: age-adjusted Charlson comorbidity index; ASA: American Society of Anesthesiologists; RBC: red blood cell; EBL: estimated blood loss

**Fig. 3:**
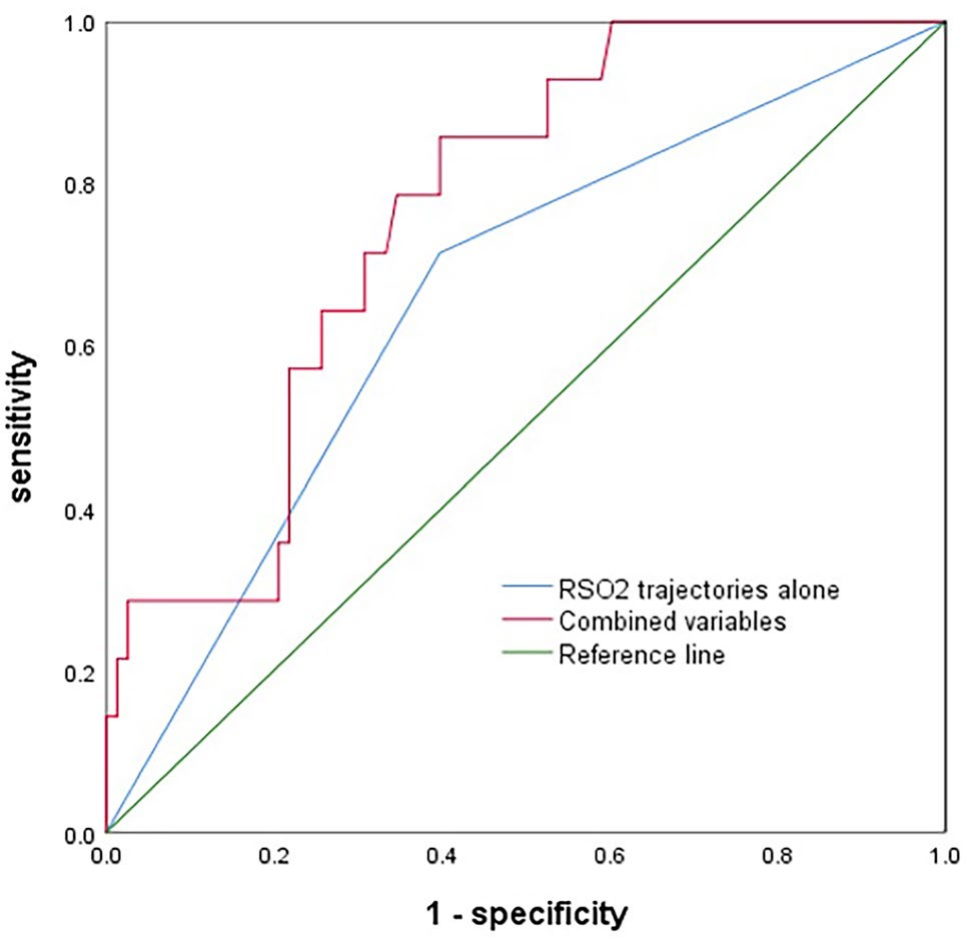
Receiver Operating Characteristic (ROC) curve of the trajectory prediction model with renal tissue RSO_2_ trajectories alone and combined variables

As shown in Table 2, the intraoperative "high-downward" RSO_2_ trajectory was associated with a higher risk of postoperative AKI (*p* = 0.028). Postoperative AKI occurred in 24.4% (10 in 41) of the patients who had a " high-downward" RSO_2_ trajectory and 7.8% (4 in 51) of the patients who had a consistently high RSO_2_ trajectory. In univariate analysis, the "high-downward" RSO2 trajectory was associated with a higher risk of postoperative AKI (unadjusted OR, 3.790; 95% CI 1.091–13.164; *p* = 0.036). No other significant associations between the clinical factors and AKI were detected from univariate logistic regression analysis. The association between the RSO_2_ trajectories and AKI remained significant after further adjustments for pre- and intraoperative confounders (adjusted OR, 3.973; 95% CI 1.020–15.478; *p* = 0.047) (Supplementary Table 1). Also, the postoperative complications were compared between the distinct trajectory groups, and the patients in the "high-downward" RSO_2_ trajectories suffered more from postoperative infection, prolonged mechanical ventilation, surgery-related complications, and ICU admission although the differences were not statistically significant due to small samples. Additionally, they had longer hospitalization and more in-hospital mortality (Table 3). Receiver Operating Characteristic (ROC) curve of the trajectory prediction model was drawn (Fig. 3). Predictive performance in identifying AKI had 71.4% sensitivity and 60.3% specificity for RSO_2_ trajectories alone (AUC: 0.658), and 85.7% sensitivity and 60.3% specificity for combined use of RSO_2_ trajectories, age, history of hypertension, and intraoperative blood transfusion (AUC: 0.763). The renal tissue RSO_2_ values at hepatic occlusion period showed best predictive performance (Supplementary Table 2).

**Table 2:** Baseline characteristics and intraoperative data in patients with or without AKI

Characteristics	Non-AKI (*n* = 78)	AKI (*n* = 14)	*t/z*/*χ*^2^	*p*-value
Renal tissue RSO_2_ trajectories, *n* (%)			4.824	0.028
High-downward group	31 (39.7)	10 (71.4)		
Consistently-high group	47 (60.3)	4 (28.6)		
Age ≥ 70 y, *n* (%)	16 (20.5)	6 (42.9)	2.145	0.143
Gender, *n* (%)			0.231	0.630
Male	59 (75.6)	12 (85.7)		
Female	19 (24.4)	2 (14.3)		
BMI ≥ 24 kg/m^2^, *n* (%)	44 (56.4)	8 (57.1)	0.003	0.959
ACCI, median (IQR)	8 (7, 9)	8 (7, 8.8)	0.102	0.919
Hypertension, *n* (%)	29 (37.2)	8 (57.1)	1.967	0.161
Diabetes, *n* (%)	19 (24.4)	4 (28.6)	0	1.000
ASA, *n* (%)			–	1.000
I	1 (1.3)	0 (0)		
II	64 (82.1)	12 (85.7)		
III	13 (16.7)	2 (14.3)		
Serum creatinine (μmol/L), mean ± SD	72 ± 13.7	72.3 ± 14.3	0.085	0.932
RBC count (10^12^/L), mean ± SD	4.4 ± 0.5	4.1 ± 0.7	1.866	0.065
Surgery duration (min), median (IQR)	258 (228.5, 343.8)	317.5 (240, 370)	0.669	0.504
Colloid volume (mL), median (IQR)	500 (500, 1000)	500 (500, 1000)	0.468	0.640
Crystal volume (mL), median (IQR)	2100 (1700, 2600)	2250 (2100, 2600)	1.283	0.200
Urine volume (mL), median (IQR)	300 (162.5, 600)	400 (225, 637.5)	0.552	0.581
EBL (mL), median (IQR)	200 (100, 300)	250 (112.5, 387.5)	0.645	0.519

RSO_2_: regional oxygen saturation; AKI: acute kidney injury; BMI: body mass index; ACCI: age-adjusted Charlson comorbidity index; ASA: American Society of Anesthesiologists; RBC: red blood cell; EBL: estimated blood loss

**Table 3:** Comparison of postoperative adverse complications during hospitalization between distinct renal tissue RSO_2_ trajectories

Characteristics	"High-downward" group (*n* = 41)	"Consistently-high" group (*n* = 51)	*p*-value
ICU admission, *n* (%)	11 (26.8%)	7 (13.7%)	0.115
Infection, *n* (%)	1 (2.4%)	0 (0%)	0.446
Prolonged mechanical ventilation, *n* (%)	1 (2.4%)	0 (0%)	0.446
Surgery-related complications, *n* (%)	2 (4.9%)	0 (0%)	0.196
Hydrothorax and ascites, *n* (%)	1 (2.4%)	2 (3.9%)	1.000
AKI, *n* (%)	12 (29.3%)	6 (11.8%)	0.035
Length of hospital (day), median (IQR)	20 (14,24)	17 (14,20)	0.096
In-hospital mortality, *n* (%)	1 (2.4%)	0 (0%)	0.446

RSO_2_: regional oxygen saturation; ICU: intensive care unit; AKI: acute kidney injury

## Discussion

To the best of our knowledge, this investigation was the first study to discover intraoperative renal tissue RSO_2_ dynamic change trajectories. Older patients who fell into the “high-downward” trajectory of renal tissue RSO_2_ exhibited an increased subsequent risk of AKI, and a good prediction accuracy of AKI can be achieved by trajectories. Furthermore, through distinct trajectories, the minimum level of renal tissue RSO_2_ was found during the period of hepatic occlusion, indicating a "time of concern" to guide interventions.

Although there is a high incidence of morbidity leading to adverse outcomes, an understanding of mechanisms underlying the complex pathophysiology of AKI is lacking, and effective prediction and advanced treatment are absent. The current diagnostic criteria are mainly based on serum creatine level, indicating kidney dysfunction [16]. However, a detectable increase in the serum creatinine level does not immediately follow the onset of AKI, and therefore, serum creatinine level alone is of limited value for early identification of the risk for AKI [17]. Biomarkers of the initial stage of renal injury, such as neutrophil gelatinase-associated lipocalin (NGAL), cystatin C, and kidney injury molecule 1, can detect AKI at the early stage [18], but their levels cannot be measured continuously to monitor the renal function in real-time. Recently, the application of NIRS has gained increasing attention, with the advantage of being noninvasive, highly sensitive, and real-time monitoring [19]. Previous studies demonstrated good agreements with invasively measured renal vein oxygen saturation (SrvO_2_) and SpO_2_ [20]. Further evidence was presented on the relationship between renal oxygen desaturation and adverse renal outcomes. Owens et al. [21] found that low RSO_2_ (defined as an absolute value of < 50% lasting for at least 2 h) was significantly correlated to higher serum creatine levels and increased risk of AKI. Yu et al. [14] concluded that older patients with RSO_2_ desaturation defined as at least 20% relative decline from the baseline regardless of duration, had a threefold risk of postoperative AKI compared with the patients without RSO_2_ desaturation. The NIRS is considered a promising method in identifying prognostic predictors of postoperative AKI for early diagnosis [22].

The trajectory optimization method for handling continuous data has a great advantage to reveal its dynamics. There is accumulating evidence that several types of blood pressure trajectories are important predictors of unfavorable outcomes [23, 24]. Tanaka’s longitudinal study identified developmental blood pressure trajectory classes among patients with acute intracerebral hemorrhage [23]. Four trajectory groups were observed, and the high-to-low group was associated with an increased risk of adverse outcomes and death. Lin’s research investigated that “high-downward-upward” blood pressure trajectory was identified as a risk factor for new-onset chronic kidney disease [25]. As a novel indicator, the trajectory model was applied for revealing RSO_2_ changes. The trend was developed to depict RSO_2_ changes in preterm neonates in Caliskan’s study [26], but it differed from the “trajectory model”. To date, there have been no reports of renal tissue RSO_2_ using the trajectory method among older patients undergoing open hepatectomy. The trajectories of RSO_2_ during the intraoperative period classified patients into several distinct subtypes and provided information on patients’ outcomes.

Combining the monitoring device and the innovative analysis approach, our study indicated that patients whose intraoperative renal tissue RSO_2_ was of a “high-downward” trajectory were at higher risk for AKI and other complications. Meanwhile, the severe imbalance of renal autoregulation may occur during the hepatic occlusion period with the lowest renal tissue RSO_2_ level. We speculate the following mechanism to explain. The kidney is one of the most important organs and has the capacity for autoregulation and maintaining homeostasis at least to some extent. When blood pressure level continuously fluctuates at a certain range, renal perfusion and oxygenation are unaffected. However, aging patients have impaired autoregulatory capacity of kidneys, and renal hemodynamics is strongly affected during the hepatic inflow occlusion, indicating that slight changes could potentiate a significant reduction in the renal perfusion, thus resulting in regulatory dysfunction as well as renal ischemia and hypoxia, which are the key factors for the development of AKI. Though the relationship between low renal tissue RSO_2_ and increased risk of AKI has been recognized in several studies [27–29], the dynamic changes of in patients at risk of AKI have not yet been investigated. Through observing the renal tissue RSO_2_ trajectories, patients with renal autoregulatory dysfunction can be identified with a high level of sensitivity. The present study found that patients with a “high-downward” trajectory with remarkably volatile changes of renal tissue RSO_2_ were more prone to impaired renal autoregulatory ability as well as hemodynamic instability whereas patients with a stable trajectory had robust regulatory functions. The “high-downward” trajectory can be considered a risk profile, indicating kidney dysfunction, and consequent postoperative AKI. Furthermore, finding lowest level was also key to take more attention of renal hemodynamics and function especially during hepatic occlusion in order to achieve early intervention. In general, the results suggested that renal tissue RSO_2_ monitoring in high-risk surgical populations could be useful in the early recognition of vulnerable renal blood regulatory capacity and secondary hypoxia and ischemia. When an apparent downward trend is observed in the renal tissue RSO_2_ during the operation or hepatic inflow occlusion progression, early intervention can be carried out immediately to preserve the renal function and avoid unwanted poor prognosis.

The study also had several limitations. Since this was an observational study, it was limited to retrospective analysis and a small sample size. And though the key time point was raised, the intervention thresholds and effective measurements remains unclear and need future prospective interventional studies. Second, the renal tissue oxygen saturation monitoring was not suitable for obese patients due to limited skin-to-kidney distance so it could not be generalized to a wider population. Third, the technique was unable to discriminate the renal cortex and inner and outer medulla which had different oxygen partial pressure values.

In conclusion, our study identified two distinct renal oxygen saturation change trajectories with a reasonably good prediction effect for AKI in older patients who underwent open liver resection. Furthermore, the hepatic occlusion period can be considered as a "time of concern" due to the minimal level of renal tissue RSO_2_. The preliminary exploration of our study may provide information for early detection, thereby preventing renal diseases.

### Supplementary Information

Below is the link to the electronic supplementary material.

Supplementary file1 (DOCX 21 KB)

## Data Availability

The data underlying this article will be shared on reasonable request to the corresponding author.
