# Supplementary material for: The relationship between trajectories of renal oxygen saturation and acute kidney injury: a prospective cohort study with a secondary analysis
Source: Aging Clin Exp Res. 2024 Feb 21;36(1):46. doi: 10.1007/s40520-024-02701-1 (PMC10881632; doi:10.1007/s40520-024-02701-1)
Supplement: Supplementary file 1 — Supplementary file1 (DOCX 21 KB) [file 40520_2024_2701_MOESM1_ESM.docx]

**Supplement**

For: Chang Liu, Xiaoxiao Wang, Wenzhu Shi et al. The relationship between trajectories of renal oxygen saturation and acute kidney injury: a prospective cohort study with a secondary analysis.

**Supplementary Table 1**. Multivariable logistic regression analysis of association between renal tissue RSO_2_ trajectories and postoperative AKI.

| Characteristics | B | SE | p | OR (95% CI) |
| --- | --- | --- | --- | --- |
| RSO_2_ trajectories (Ref: Consistently-high) |  |  |  |  |
| High-downwards | 1.379 | 0.694 | 0.047 | 3.973 (1.020, 15.478) |
| Aged ≥70 yr | 1.099 | 0.689 | 0.111 | 3.002 (0.778, 11.588) |
| Hypertension history | 1.377 | 0.690 | 0.046 | 3.961 (1.024, 15.330) |
| Intraoperative transfusion | -0.839 | 0.561 | 0.135 | 0.432 (0.144, 1.298) |

***Abbreviation:*** RSO_2_**:** regional oxygen saturation; AKI: acute kidney injury

**Supplementary Table 2**. The prediction performance of renal tissue RSO2 in each time point to AKI.

| Time point | Sensitivity | Specificity | ROC |
| --- | --- | --- | --- |
| T1 | 63.3% | 64.3% | 59.4% |
| T2 | 87.5% | 44.8% | 61.4% |
| T3 | 89.9% | 42.9% | 64.0% |
| T4 | 88.6% | 57.1% | 68.4% |
| T5 | 91.1% | 42.9% | 66.5% |
| T6 | 91.1% | 50.0% | 68.1% |
| T7 | 88.6% | 50.0% | 68.5% |
| T8 | 54.4% | 78.6% | 67.7% |
| T9 | 69.6% | 57.1% | 62.5% |
| T10 | 79.7% | 50.0% | 64.9% |
| T11 | 65.6% | 72.9% | 66.5% |
| T12 | 64.6% | 75.7% | 64.1% |
